# Supplementary material for: Complex‐centric proteome profiling by SEC‐SWATH‐MS
Source: Mol Syst Biol. 2019 Jan 14;15(1):e8438. doi: 10.15252/msb.20188438 (PMC6346213; doi:10.15252/msb.20188438)
Supplement: Supplementary file 8 — Dataset EV7 [file MSB-15-e8438-s008.zip › feature_plots_string/P13073.pdf]

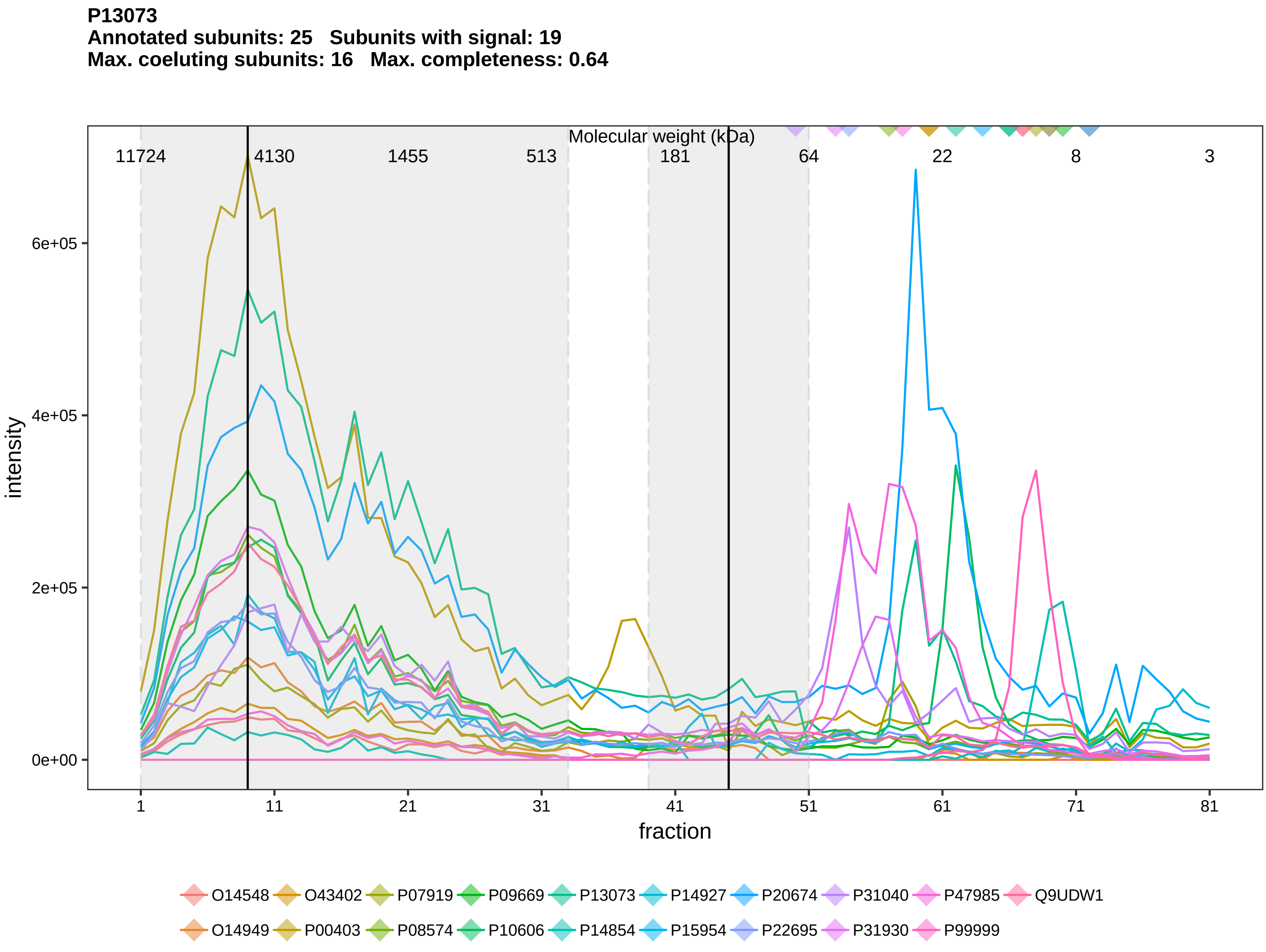
P13073
Annotated subunits: 25 Subunits with signal: 19
Max. coeluting subunits: 16 Max. completeness: 0.64
Molecular weight (kDa)
11724
4130
1455
513
181
64
22
8
3
intensity
6e+05
4e+05
2e+05
0e+00
fraction
1
11
21
31
41
51
61
71
81
O14548
O43402
P07919
P09669
P13073
P14927
P20674
P31040
P47985
Q9UDW1
O14949
P00403
P08574
P10606
P14854
P15954
P22695
P31930
P99999
